# The global switch from trivalent oral polio vaccine (tOPV) to bivalent oral polio vaccine (bOPV): facts, experiences and lessons learned from the south-south zone; Nigeria, April 2016

**DOI:** 10.1186/s12879-018-2963-6

**Published:** 2018-01-27

**Authors:** Bassey Enya Bassey, Fiona Braka, Rui Gama Vaz, William Komakech, Sylvester Toritseju Maleghemi, Richard Koko, Thompson Igbu, Faith Ireye, Sylvester Agwai, Godwin Ubong Akpan, Sisay Gashu Tegegne, Abdul-Aziz Garba Mohammed, Angela Okocha-Ejeko

**Affiliations:** World Health organization (WHO) Nigeria Country office, UN House, plot 617/618, Diplomatic Drive, Central Business District, Abuja, Garki PMB 2861 Nigeria

**Keywords:** tOPV, bOPV, Switch

## Abstract

**Background:**

The globally synchronized switch from trivalent Oral Polio Vaccine (tOPV) to bivalent Oral Polio Vaccine (bOPV) took place in Nigeria on April 18th 2016. The country is divided into six geopolitical zones. This study reports the experiences and lessons learned from the switch process in the six states that make up Nigeria’s south-south geopolitical zone.

**Methods:**

This was a descriptive retrospective review of Nigeria’s switch plan and structures used for implementing the tOPV-bOPV switch in the south-south zone. Nigeria’s National Polio Emergency Operation Centre (NPEOC) protocols, global guidelines and reports from switch supervisors during the switch were used to provide background information for this study. Quantitative data were derived from reviewing switch monitoring and validation documents as submitted to the NPEOC

**Results:**

The switch process took place in all 3078 Health Facilities (HFs) and 123 Local Government Areas (LGAs) that make up the six states in the zone. A total of $139,430 was used for this process. The ‘healthcare personnel’ component received the highest budgetary allocation (59%) followed by the ‘logistics’ component (18%).

Akwa Ibom state was allocated the highest number of healthcare personnel and hence received the most budgetary allocation compared to the six states (total healthcare personnel = 458, total budgetary allocation = $17,428).

Validation of the switch process revealed that eight HFs in Bayelsa, Cross-River, Edo and Rivers states still possessed tOPV in cold-chain while six HFs in Cross-River and Rivers states had tOPV out of cold-chain but without the ‘do not use’ sticker.

Akwa-Ibom was the only state in the zone to have bOPV and Inactivated Polio Vaccine (IPV) available in all its HFs monitored.

**Conclusion:**

The Nigerian tOPV-bOPV switch was successful. For future Oral Polio Vaccine (OPV) withdrawals, implementation of the switch plan would be more feasible with an earlier dissemination of funds from global donor organizations, which would greatly aid timely planning and preparations. Increased budgetary allocation to the ‘logistics’ component to accommodate unexpected hikes in transportation prices and the general inefficiencies with power supply in the country is also advised.

## Background

The launch of the Global Polio Eradication Initiative (GPEI) by the World Health Assembly (WHA) in 1988 set the stage for global polio eradication [1]. This led to the dramatic decline in the number of poliomyelitis cases due to wild poliovirus (WPV) from an estimated 350,000 cases in 1988 to about 37 cases in 2016 [2–4]. The transmission of WPV has been limited to just three countries, Afghanistan, Pakistan and Nigeria [5]. Reports from 2016 showed that Pakistan and Afghanistan reported 20 and 13 WPV type1 (WPV1) cases respectively [5–7].Nigeria was readmitted to the list of polio endemic countries following the detection of four WPV1 cases and a single case of circulating vaccine-derived poliovirus type 2 (cVDPV2) in August 2016 [8]. No new cases of WPV have been reported as Nigeria continues to implement emergency outbreak response in response to the detected WPV1 strain and cVDPV2 strains affecting the country [6].

Despite polio eradication setbacks in Nigeria, the GPEI has made significant efforts towards polio eradication through the use of Oral Polio Vaccine (OPV) i.e. trivalent Oral Polio Vaccine (tOPV), which contains Sabin strains of all three poliovirus serotypes (i.e. OPV 1,2 and 3) [9]. The use of this vaccine has led to the eradication of WPV type 2 (WPV2) [10]. The last known case of WPV2 dates back to 1999 in northern India and WPV2 was declared eradicated by the Global Certification Commission (GCC) in 2015 [10, 11]. The last case of WPV type 3 (WPV3) dates back to 2012 in Nigeria [12]. Four of the six World Health Organization (WHO) regions have also been certified as polio-free [13].

However, attenuated polioviruses in OPV can undergo genetic changes during replication, and in communities with low vaccination coverage, can result in vaccine-derived polioviruses (VDPVs) [14]. These can cause paralytic polio indistinguishable from the disease caused by WPVs [12]. These VDPVs can further acquire transmissibility and become circulating VDPVs (cVDPVs) [3].

In response to the risk associated with tOPV use, WHO Strategic Advisory Group of Experts on immunization (SAGE), together with the GPEI developed a comprehensive long-term plan called the Polio Eradication & Endgame Strategic Plan (PEESP), 2013–2018 [15, 16]. Endorsed in 2013 by the 66th WHA, this plan consists of four principal objectives that address the eradication of all polio diseases whether caused by WPVs or cVDPVs [17].

Since the eradication of WPV2, cVDPV2s due to OPV2 have for accounted for 94% of the 721 poliomyelitis cases caused by cVDPVs detected between January 2006 and May 2016 [14]. To stop further risks of cVDPV2s, SAGE reaffirmed the need for the switch from tOPV to bivalent Oral Polio Vaccine (bOPV), which contains Sabin serotypes 1 and 3 strains (i.e. OPV 1and 3) [18, 19] This decision was reached after reviewing the epidemiology of cVDPV2s and all criteria necessary for the switch in line with the objectives of the PEESP in October 2015 [18–20]. But, one country cannot just stop tOPV use if other countries that may export OPV2-related viruses continue to use tOPV. This is because, as its population becomes more susceptible, imported OPV2-related viruses can easily establish sustained circulation and develop into cVDPV2s [21, 22]. As a result, the switch was carried out in a globally synchronized manner between 17th April and 1st May to prevent the risk of exporting OPV2-related viruses (i.e. polioviruses related to the serotype 2 strain in tOPV) from the countries using tOPV to other neighbouring areas that have stopped tOPV use [14, 21, 22].

However, the cessation of tOPV use also carries some risk for facilitating the spread of undetected or newly emergent cVDPV2s among persons without immunity to serotype 2 polioviruses after the switch. [12] To stop the spread of existing cVDPV2s before the switch and to prevent post switch outbreaks, population immunity to serotype 2 polioviruses at the time of the switch were to be boosted with Supplementary Immunization Activities (SIAs) using tOPV along with the introduction of at least one dose of Inactivated Polio Vaccine (IPV) to the Routine Immunization (RI) system [14]. The introduction of IPV will provide vaccinated children born after the switch with individual protection from poliomyelitis caused by serotype 2 polioviruses [17].

Nigeria complied with these requirements when she carried out SIAs across the country using tOPV for 3 weeks before the switch to boost immunity against serotype 2 polioviruses. This was coupled with the phased introduction of IPV into the country’s RI schedule well over 6 months before the April/May 2016 global switch period.

The tOPV-bOPV switch took place in Nigeria on the 18th of April 2016. This study reports the experiences and lessons learned from the tOPV-bOPV switch process in south-south zone of Nigeria.

## Methods

### Study location

**Fig. 1:**
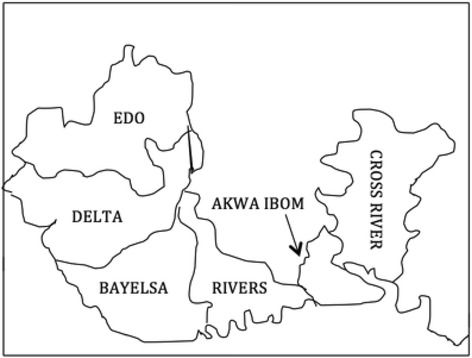
Map showing the states in the south-south zone of Nigeria

Nigeria is divided into six geopolitical zones namely: north-east, north-west, north-central, south-south, south-east and south-west. This study documents the switch from tOPV to bOPV in the south-south zone of Nigeria. This zone comprises of six states and 123 Local Government Areas (LGAs) namely: Akwa Ibom (31), Bayelsa (8), Cross-River (18), Edo (18), Delta (25) and Rivers (23). The zone is commonly referred to as the Niger Delta region of the country because it is located (in the southern part of Nigeria) at the point where the ‘Y’ tail the river Niger joins the Atlantic Ocean through the Gulf of Guinea. According to the 2006 census, the population of the south-south region is estimated at 24.6 million people [23]. Figure 1 shows the states in the south-south zone of Nigeria.

### Study design

This was a descriptive retrospective review of Nigeria’s switch plan and structures used for implementing the tOPV-bOPV switch in the south-south zone of the country. Nigeria’s National Polio Emergency Operation Centre (NPEOC) protocols and global guidelines together with reports from switch supervisors during the switch were used to provide background information for this study. Quantitative data were derived from reviewing switch monitoring and validation documents as submitted to the NPEOC.

The switch was conducted in such a way that most activities occurred at the Health Facility (HF) level. Switch activities were coordinated at different levels (LGAs, state) but final coordination was done at the national level. Clear timelines were given for the implementation of each switch activity and these were monitored using standardized dashboards. The key activities conducted were: establishment of switch committees, training of health workers, sensitization of stakeholders, data management and inventory, production and distribution of data tools and bOPV vials to HFs, switch implementation, switch monitoring and validation, the ‘sweep process’ and tOPV destruction. The ‘sweep process’ was a systematic search conducted in all HFs in an LGA where tOPV vials were found to ensure the complete withdrawal of residual vaccines in that LGA.

### Establishment of switch committees

The Nigerian government established switch support committees at the three levels of government i.e., national, state, and LGA. These committees were responsible for implementing the activities of the national switch plan.

Switch committees were established in the states and LGAs on the 12th and 28th of February 2016 respectively. The switch was structured along the polio eradication structure of the NPEOC [24]. The Incident Manager (IM) of the NPEOC was the overall coordinator of the switch process.

Key activities conducted by these committees included coordination, development of switch plan and budget based on global guidelines, adaptation of guidelines, setting up operations centre, training, monitoring and mapping of human resource requirements, supervision and validation. The committees consisted of staff from Ministry of Health (MOH), National Agency for Food and Drug Administration and Control (NAFDAC), and international partners involved in immunization. The switch committees had a schedule of activities and initially met weekly but as the date of the switch approached, the meetings were daily to identify issues and proffer solutions. There were also conference calls between different levels (National and state, state and LGA) to obtain real time data on challenges and give solutions.

Subcommittees on logistics, communications and mobilization, and data management were also established to monitor bOPV arrival and receipts, adaptation of tools and development of advocacy kits. The NPEOC also assigned Monitoring and Accountability (M&A) officers to state clusters to follow up on the implementation of key activities. These officers provided regular reports to the NPEOC. The data team generated monitoring data and flagged issues of concern to the switch committees, which guided their resolution.

### Training

Health workers for the switch were trained alongside the supplemental immunization campaign training conducted in February and March 2016. The training explained the importance of the switch and the processes involved in conducting actual switch activities. The importance of timeliness and completeness of each stage of the switch process was also stressed during the training. Other avenues used to train health workers on the switch process were: the monthly state-level Expanded Programme on Immunization (EPI) and surveillance review meetings, heads of health facilities meetings and professional association meetings.

### Sensitizing stakeholders

Most trainings and meetings focused on the health workers in the public sector. However, it became imperative to meet with private sector health workers, pharmacists, pharmaceutical firms and other health stakeholders on the importance of the tOPV switch because they partner with the government in the provision of quality immunization services.

This was done via letters and one-on-one meetings using line-lists of potential persons or facilities that could have OPV. At such forums, the issues discussed were: the importance of the switch and tOPV retrieval methods. Assurances were also given to the HFs and vaccine cold stores that all tOPVs retrieved would be replaced with bOPVs without any financial losses to them.

### Data management and inventory

A vaccine management tool was used to monitor tOPV stock. Where there was overstocking, vaccines were redistributed to other facilities to avoid wastage. Activities such as mopping-up campaigns with tOPV were conducted. Physical counts were done regularly at all HFs (that had storage capacity), LGA and state cold stores to track tOPV stock levels. An electronic dashboard was used to track the implementation status of these activities. Progress made was shared weekly with the NPEOC.

### Production and distribution of data tools, bOPV vaccines and other materials

All states in the zone received bOPV vials, along with data tools, stickers and hazard bags from the national level. Arrangements were made at the state level to procure other needed materials. The materials were moved to the LGAs on the 12th of April 2016 and finally to the HFs.

### Switch implementation

The switch was implemented using the reverse SIA approach and it involved all health workers in the facilities that stored tOPV. The LGAs ensured that HFs were open on the 16th and 17th of April for the collection of all tOPVs. The vaccines were collected in hazardous bags with stickers clearly stating that they were tOPVs up for withdrawal. The ward focal persons from each ward picked up the tOPV vials from the HFs and replaced them with the same quantity of bOPV. Appropriate documentation of vaccine pick-up/replacement was ensured. A search was also conducted to ensure that tOPV vials were not left behind in the facilities.

Supervisory plans were drawn and senior monitors/supervisors were used to ensure compliance with the laid down procedures. They also ensured that vaccines were not left behind in the facilities. All collected tOPV vials were moved to designated storage places. This was done using the mechanism put in place by the LGA cold stores for vaccine movement. Vaccines were moved from the LGAs to identified storage spots for destruction in the state capitals.

Before tOPV destruction and monitoring by independent validators/monitors, senior switch supervisors in the state conducted further spot checks to assess the quality of the switch and ensured that bOPVs were given to HFs to prevent the disruption of immunization activities. They also ensured that the new data tools were being used to capture bOPV use.

### Switch monitoring and validation

Fifty-four validators were sent from the national level to all states in the south-south zone to verify the quality of the tOPV-bOPV switch in each LGA. These validators were independent health workers specifically hired for the switch. Each validator worked for 7 days to verify the quality of the entire switch process. Using standard data tools, they were mandated to visit 10% of all HFs in the LGA and report on several aspects of the switch process.

### Sweep process

The ‘sweep process’ was conducted in LGAs where validators found tOPV vials after the withdrawal was done. Switch validators assessed 10% of the HFs in each LGA. If tOPV vials were found in any of the HFs visited, an additional 5% of the HFs in the LGA would be visited. Where there were more tOPV vials found, all the HFs in the LGA were then recommended for a sweep, i.e., all the HFs in the LGA would be visited by the validators to ensure the complete removal of leftover tOPV vials.

### tOPV destruction

This was the final stage of the switch process, which was done centrally in all states. tOPV destruction was supervised by the state tOPV switch committee and was certified by NAFDAC. The method chosen was the ‘boil and bury’ method where all the vaccines retrieved were boiled in huge drums and buried in dug pits. The process was properly documented and all the vaccines destroyed were accounted for by the LGAs and state.

## Results

The tOPV-bOPV switch process was a critical step in the polio eradication endgame strategy. As such, adequate plans and budgetary arrangements were made to successfully implement switch activities in the 3078 HFs spread across 123 LGAs in the south-south zone of Nigeria (Table 1).

**Table 1:** Distribution of Health Facilities in the south-south zone involved in the tOPV - bOPV switch process in Nigeria, 2016

State Name	Total LGAs	Total Health Facilities
Akwa Ibom	31	513
Bayelsa	8	170
Cross-River	18	644
Delta	25	560
Edo	18	574
Rivers	23	617
Total	123	3078

Figure 1 shows the budgetary implication of the tOPV- bOPV switch in the south-south zone. A total of $139,430 was allocated and used for the switch. The ‘healthcare personnel/human resource’ component got the highest budgetary allocation (59%). The ‘logistics and waste management’ component followed this with 18%. The least budgetary allocations were given to the ‘training materials’ (2%) and ‘training and meetings’ (3%) component.

Healthcare personnel (Nurses, community health officers, community health extension workers) played an important role in facilitating and ensuring the success of the switch program. Table 2 shows the distribution of healthcare personnel involved in the tOPV-bOPV switch and the budgetary allocations used to fund their activities.

**Table 2:** Distribution of health care personnel involved in the tOPV-bOPV switch, south-south zone, Nigeria 2016

State name	Number of personnel	Amount allocated ($)	% Amount allocated
Akwa-Ibom	458	17,428	21
Bayelsa	143	5318	6
Cross-River	274	14,075	17
Delta	374	15,747	19
Edo	270	13,235	16
Rivers	417	16,820	20
	1963	82,622	

A total of 1963 healthcare personnel were used to facilitate the switch process and $82,622 was used to finance their activities.

Akwa-Ibom state used the most (458) healthcare personnel and as such, received the highest budgetary allocation of 21%. Rivers state closely followed this with 417 healthcare personnel and a budgetary allocation of 20%.

Bayelsa state utilized the least healthcare personnel (143) and as such, only got 6% of the overall budgetary total. Edo and Cross-River states followed this with 270 and 274 healthcare personnel respectively. They received 16% and 17% respectively of the zone’s budgetary allocation.

Table 3 shows the quantities of tOPV withdrawn, bOPV replaced and IPV available across the HFs in the south-south zone during the switch validation process.

**Table 3:** Quantity analysis of tOPV withdrawn, IPV available and bOPV replaced across HFs in the south-south zone during the switch validation process

Zone/state name	HFs monitored	HF with tOPV in cold-chain. (%)	HF with tOPV out of cold-chain without “do not use sticker” (%)	HF with IPV available (%)	HF with bOPV replaced (%)	No. Of tOPV vials withdrawn during the switch validation process.
South-south zone	391	8 (2)	6 (2)	131 (34)	205 (52)	18,701
Akwa-Ibom	39	0 (0)	0 (0)	39 (100)	39 (100)	0
Bayelsa	26	2 (25)	0 (0)	18 (69)	13 (50)	851
Cross-River	118	4 (50)	1 (17)	14 (12)	65 (55)	10,321
Delta	77	0 (0)	0 (0)	8 (10)	28 (36)	7
Edo	72	1 (13)	0 (0)	26 (36)	19 (26)	4913
Rivers	59	1 (13)	5 (83)	26 (44)	41 (70)	2609

In the south-south zone, 391 HFs were monitored and 2% of them had tOPV within active cold-chain. The states with their respective HFs implicated were: Bayelsa (2), Cross-River (4), Edo (1) and Rivers (1). Also, 2% of the total HFs monitored had tOPV out of cold-chain but without the ‘do not use sticker’. The states and their corresponding HFs implicated were Cross-river (1) and Rivers (5). However, 205 HFs had bOPV replaced and IPV was available in 131 HFs. In total, 18,701 tOPV vials were withdrawn from this zone during the switch validation process.

Vaccine withdrawal activities across the different administrative levels of the south-south zone were illustrated in Table 4. Each state in the zone has at least one cold store located in its state capital for administrative purposes and vaccine distribution to smaller cold stores strategically located in the LGAs of the state (i.e. at least one cold store per LGA). The cold stores at both the state and LGA administrative levels were responsible for the supply of bOPV and withdrawal of tOPV vials from all the HFs in the country for the switch process.

**Table 4:** Vaccine withdrawal and replacement activities across different administrative levels in the south-south zone for the Nigerian 2016 tOPV - bOPV switch

South-south Zone/states	No. Of stores monitored	Stores with tOPV in Cold-chain (%)	Stores with tOPV out of cold-chain without “do not use sticker”	Stores with bOPV replaced (%)	Stores with IPV available (%)	No. Of vials disposed or pending disposal
Sub-National/ State Administrative Level						
Akwa-Ibom	1	0(0)	0(0)	1 (100)	1(100)	217,780
Bayelsa	1	0(0)	0(0)	1(100)	1 (100)	22,000
Cross-River	1	0(0)	0(0)	1 (100)	0 (0)	65,000
Delta	2	0(0)	0(0)	2 (100)	1 (50)	0
Edo	1	0(0)	0(0)	1 (100)	1 (100)	294,078
Rivers	1	0(0)	0(0)	1(100)	0 (0)	48
Local Government Area (LGA) Administrative Level						
Akwa-Ibom	31	0(0)	0(0)	31(100)	31(100)	157,079
Bayelsa	8	0(0)	0(0)	8(100)	8(100)	78,125
Cross-River	18	2(11)	1(6)	18(100)	3(17)	59,541
Delta	24	1(4)	1 (4)	22(91)	3(13)	50,851
Edo	15	0(0)	0(0)	14(93)	12(80)	41,731
Rivers	22	0(0)	5(23)	22(100)	18(82)	90,806

At the state administrative level, there were no tOPVs out of cold-chain lacking the ‘Do not use’ sticker. All the state cold stores in the zone had also completely replaced tOPV with bOPV stocks. However, only 50% of the required IPV stock was available at the Delta state cold stores.

At the LGA administrative level, tOPV in cold-chain was found in 11% of the 18 stores in Cross-River and 4% of the 24 stores in Delta state. About 6% of the stores in Cross-River state, 4% of the stores in Delta state and 23% of the store in Rivers states had tOPV out of cold-chain but without the ‘do not use’ sticker. bOPV was completely replaced in all the LGA cold stores in the zone except for the Delta and Edo state LGA cold stores that had 91% and 93% availability for bOPV in their cold stores.

IPV was available in only 17% of the LGA cold stores in Cross-River state, 13% of the LGA cold stores in Delta state, 80% of the LGA cold stores in Edo state and 82% of the LGA cold stores in Rivers state. The LGA cold stores in Akwa Ibom and Bayelsa states had 100% availability for IPV.

Table 5 shows the states, LGAs and HFs where the ‘sweep process’ was conducted. The ‘sweep process’ was a systematic search conducted in all HFs and vaccine cold stores to ensure the complete withdrawal of all tOPVs. This procedure was conducted in LGAs where switch validators found tOPV vials.

**Table 5:** Distribution of States, LGAs and HFs where the ‘sweep process’ was conducted in the south-south zone; Nigeria, 2016

State Name	Total LGAs in state	No of sweep LGAs (%)	Total HFs in state	No of sweep HFs (%)
Bayelsa	8	1 (12.5)	170	2 (1.2)
Cross River	18	1 (5.5)	644	3 (0.5)
Total	26	2 (7.7)	814	5 (19)

The ‘sweep process’ was ordered in two states: Bayelsa and Cross-River states. These states consist of 26 LGAs (Bayelsa: 8, Cross River: 18) and 814 HFs (Bayelsa: 170, Cross River: 644).

The ‘sweep process’ was conducted in two of the 26 LGAs in both states (one LGA per state). Switch validators visited six HFs (Bayelsa: 2, Cross River: 4) in these LGAs during the ‘sweep process’.

## Discussion

The globally synchronized tOPV-bOPV switch process was a key milestone in the efforts to eradicate poliomyelitis because it marked the last step needed to eliminate all serotype 2 polioviruses. As of June 2017, two separate cases of cVDPV2 possibly associated with inadvertent use of tOPV had been confirmed in the Democratic Republic of Congo (DRC) [25]. This event further demonstrates that the risk from tOPV use is real and makes it even more important to ensure the complete removal of all tOPV from the vaccine supply chains in all countries.

**Fig. 2:**
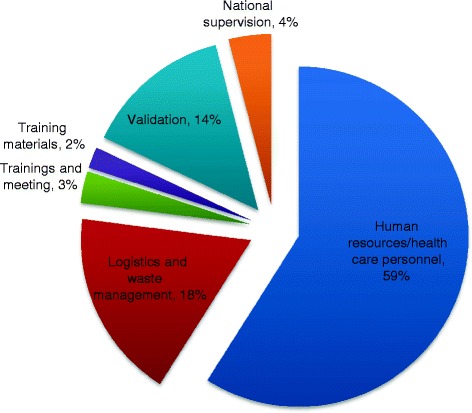
Budgetary Implication of the tOPV-bOPV switch in the south-south zone, Nigeria 2016

The tOPV-bOPV switch process took place in Nigeria on the 18th of April 2016. The switch was conducted in all 3078 HFs and 123 LGAs that make up the six states in the south-south zone. (Table 1) The zone used about $139,430 to facilitate the entire switch process. The human resource/healthcare personnel component of the budget received the largest budgetary allocation of 59%. (Figure 2) This was not surprising because health workers played a crucial role in facilitating and ensuring the success of the entire switch process.

Akwa Ibom state utilized the highest number of health care personnel and hence received the highest budgetary allocation of 21%. This was probably because it had the highest number of LGAs in comparison to the other states in the zone. Bayelsa state was allocated the least amount of money and health care personnel probably for the same reason (Table 2).

In general, the presence of more healthcare personnel in Akwa Ibom state could have contributed to the reason why this state performed better in terms of completeness of tOPV withdrawal from the LGAs and HFs, and supply of bOPV and IPV. It was the only state in the zone to have no tOPV out of cold-chain lacking the ‘do not use’ sticker (Table 3). It was also the only state in the zone with 100% availability for IPV and bOPV in all its HFs monitored. The other states had vaccine shortfalls either at the cold stores in their state administrative level or at the cold stores in their LGA administrative level (Table 4).

The implication of these vaccine shortfalls is that it discourages caregivers from coming to immunise their wards against poliomyelitis and those that do come, would have to travel long distances to get to the HFs with vaccine availability. This defeats the overall purpose of RI and may even affect the polio herd immunity in such communities.

However, we believe that the nature of the vaccine supply chain within the country could have contributed to the overall shortage of bOPV and IPV in HFs monitored across the zone. The south-south zone operates a ‘Push-Pull’ vaccine transport system where the vaccines in the zonal cold stores are pushed to their respective state cold stores. From there, the vaccines are either pulled or pushed to the LGA cold stores from where the various HFs come and collect them for use. It is possible that the mismanagement of this system could have affected bOPV and IPV availability in majority of the HFs monitored.

Improper documentation could have also contributed to the vaccine shortfalls at the LGA level. Vaccines are usually given to LGAs and HFs based on consumption figures collated and submitted to the LGA cold stores, which gets submitted to the cold stores at the state level. It is possible that the LGA teams responsible failed to give accurate vaccine utilization figures and this could have affected the availability of vaccines in most parts of the country.

Results from the ‘sweep process’ showed that the sweep was conducted in two out of the six states in the zone in accordance with the implementation guideline for the tOPV-bOPV switch [26]. This required that a sweep be carried out if two HFs were found with either tOPV in cold-chain or out of cold-chain but without the ‘do not use ‘sticker in an LGA. All HFs in that LGA must then be visited and purged of any residual tOPV. The high *riverine* area to landmass ratio in Bayelsa state together with the mountainous nature of some settlements in Cross River state could have contributed to the reason why some LGAs in these states were recommended for the sweep. The terrain in these states could have discouraged the healthcare personnel responsible for the affected HFs in the LGAs from properly ensuring the complete removal of tOPV.

The Nigerian tOPV-bOPV switch was referred to as successful. However, this success didn’t come without some general challenges in the south-south zone. These challenges include the late notification of exact monies available for the switch. The budgetary process for the switch began early in the country but due to late notification of the exact level of funding available, the proposed budget of four million USD was reviewed downwards to just over two million USD. The allocated money for the budget also arrived late and this impacted negatively on the timelines allocated for some key activities such as supervision and training. Early implementation of the switch plan was also affected as the number of health workers originally engaged for the switch had to be downsized to fit into the new budget.

High cost of water transportation in hard to reach areas particularly, the riverine areas of the Niger Delta also posed a challenge considering the downsizing of the proposed budget. The unreliable nature of the Nigerian electricity sector also made it difficult to maintain cold chain and avoid vaccine exposure to heat. This put strain on the already tight budget as alternative means of power supply had to be provided to store the vaccines at both the state, LGA and HF levels.

Finally, the management of the vaccine supply chain was weak in terms of ensuring 100% availability of bOPV in HFs during the switch. The vaccine stock data were also not readily available and this negatively affected the integrity of the entire vaccine supply system. We advocate accurate record keeping by the LGA team, as this would have greatly enhanced the vaccine distribution chain across the HFs.

Even with implementation challenges, some best practices worthy of recognition were observed during this switch process. These were: the weekly teleconferences held between the national Emergency Operation Centre (EOC) and the subnational levels on the progress of the switch process, the timely dashboard analysis and real-time feedback from the health workers in the HFs, the weekly update on the switch process from the lower level (LGA) to the higher level (state/subnational) and the weekly tracking of the states with residual tOPV vials. All of these contributed to the overall success of the switch in both the zone and country.

## Conclusion

The experience gained from implementing a successful switch in the country has offered valuable insights on how to improve Nigeria’s EPI, and how to prepare for the eventual withdrawal of bOPV after polio has been eradicated.

Moving forward, we recommend the timely declaration and dissemination of the exact amount of money available for future OPV withdrawal activities by the global partners and for the timely notification of relevant stakeholders within the country of such activities. This ensures early/timely budgetary adjustments and engagements in the necessary resource mobilization activities. We also suggest that more allocation be given to the ‘logistics’ budgetary component (which covers transportation/vaccine movement) to accommodate unexpected hikes in transportation prices and inefficiencies with the country’s power supply.

## Abbreviations

bOPV: Bivalent oral polio vaccine
cVDPV: Circulating vaccine derived polio virus
cVDPV2: Circulating vaccine-derived poliovirus type 2
DRC: Democratic republic of Congo
EOC: Emergency operation centre
EPI: Expanded program on immunization
GCC: Global certification commission
GPEI: Global polio eradication initiative
HF: Health facility
IM: Incident manager
IPV: Inactivated polio vaccine
LGA: Local government area
M&A: Monitoring and accountability
MoH: Ministry of health
NAFDAC: National agency for food, drug, administration and control
NPEOC: National polio emergency operation centre
OPV: Oral polio vaccine
PEESP: Polio eradication and endgame strategy plan
RI: Routine immunization
SAGE: Strategic advisory group of experts on immunization
SIA: Supplementary immunization activities
tOPV: Trivalent oral polio vaccine
VDPV: Vaccine-derived poliovirus
WHA: World Health Assembly
WHO: World Health Organization
WPV: Wild poliovirus
WPV1: WPV type 1
WPV2: WPV type 2
WPV3: WPV type 3
